# Molecular and Immunological Characterization of Biliary Tract Cancers: A Paradigm Shift Towards a Personalized Medicine

**DOI:** 10.3390/cancers12082190

**Published:** 2020-08-06

**Authors:** Ines Malenica, Matteo Donadon, Ana Lleo

**Affiliations:** 1 Hepatobiliary Immunopathology Unit, Humanitas Clinical and Research Center-IRCCS, 20090 Pieve Emanuele, Italy; ines.malenica@humanitasresearch.it; 2 Department of Hepatobiliary and General Surgery, Humanitas Clinical and Research Center-IRCCS, 20089 Rozzano, Italy; matteo.donadon@hunimed.eu; 3 Department of Biomedical Science, Humanitas University, 20089 Rozzano, Italy; 4 Internal Medicine and Hepatology Unit, Humanitas Clinical and Research Center-IRCCS, 20089 Rozzano, Italy

**Keywords:** cholangiocarcinoma, gallbladder cancer, immune therapies, targeted therapies

## Abstract

Biliary tract cancers (BTCs) are a group of rare cancers that account for up to 3–5% of cancer patients worldwide. BTCs include cholangiocarcinoma (CCA), gallbladder cancer (GBC), and ampulla of Vater cancer (AVC). They are frequently diagnosed at an advanced stage when the disease is often found disseminated. A late diagnosis highly compromises surgery, the only potentially curative option. Current treatment regimens include a combination of chemotherapeutic drugs gemcitabine with cisplatin that have a limited efficiency since more than 50% of patients relapse in the first year. More recently, an inhibitor of fibroblast growth factor receptor 2 (FGFR2) was approved as a second-line treatment, based on the promising results from the NCT02924376 clinical trial. However, novel secondary treatment options are urgently needed. Recent molecular characterization of CCA and GBC highlighted the molecular heterogeneity, etiology, and epidemiology in BTC development and lead to the classification of the extrahepatic CCA into four types: metabolic, proliferating, mesenchymal, and immune type. Differences in the immune infiltration and tumor microenvironment (TME) have been described as well, showing that only a small subset of BTCs could be classified as an immune “hot” and targeted with the immunotherapeutic drugs. This recent evidence has opened a way to new clinical trials for BTCs, and new drug approvals are highly expected by the medical community.

## 1. Introduction: Biliary Tract Cancers

**Figure 1:**
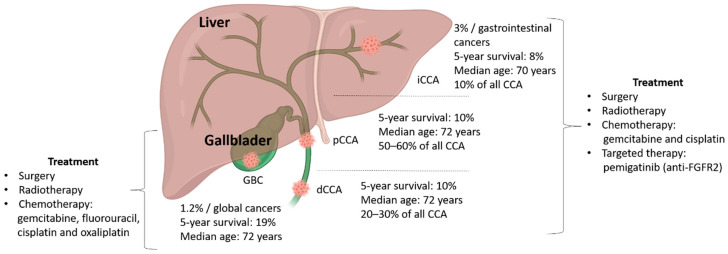
Schematic representation of the anatomic site, demographic information, and the treatment options for biliary tract cancers (BTCs). Abbreviations: dCCA: Distal cholangiocarcinoma (CCA); GBC: Gallbladder cancer; iCCA: Intrahepatic cholangiocarcinoma; pCCA: Perihilar CCA. Source: Cancer.Net.

Biliary tract cancers (BTCs) are a group of epithelial gastrointestinal malignancies that are classified based on the anatomic site inside the biliary tree, including gallbladder cancer (GBC), ampulla of Vater cancer (AVC), and cholangiocarcinoma (CCA). CCA is the most common BTC and is subclassified into intrahepatic (iCCA), perihilar (pCCA), and distal (dCCA) CCA, with the last two subtypes often classified as an extrahepatic (eCCA) CCA, due to an obsolete nomenclature [1]. BTCs are the second most common type of hepatobiliary cancers worldwide after hepatocellular cancer (HCC). They develop at a global level in both sexes, with an incidence rate of 3–5% of all diagnosed cancers (Figure 1).

BTCs are more prevalent in males than in females, and the risk of the development increases with age, with the majority of cases being diagnosed in patients over 75 years old [2]. Multiple lifestyle and diet risk factors have been described, including smoking, alcohol consumption, and aflatoxin contamination [3,4]. Moreover, medical conditions, such as obesity, cirrhosis, and chronic viral hepatitis have been implicated in the increased risk of liver cancers [5,6,7].

Despite the rare prevalence of BTCs, the mortality rate is extremely high. The disease is often identified at an advanced stage, due to the lack of specific screening programs and non-invasive diagnostic techniques. Further, the complications related to the biopsy sampling, including seeding, often lead to a delay in the diagnosis [8]. At an advanced stage, treatment options are limited, and the 5-year survival is only 2% in the case of metastasis development. A small percentage of BTC patients are eligible for the surgery when they are first diagnosed; in non-surgical patients, the use of the chemotherapeutic drugs gemcitabine and cisplatin is the standard first-line regimen [9]. Further, a recent randomized Phase 3 study demonstrated a significant benefit in overall survival (OS) and a recurrence-free survival in patients who were treated postoperatively with capecitabine, when compared to the surgery alone [10].

Over the last two decades, an increase in the incidence rate of BTCs has been observed in the Western countries [11,12] and despite the surgery and chemotherapy/radiotherapy, new therapeutic options are urgently needed. In that light, an extensive molecular characterization has been performed in multiple BTC cohorts, with some of the detected mutations/fusions identified as possible medical targets. This paradigm shift towards a personalized, molecular-based therapy opened new options and brought new hopes in cancer treatment, including BTCs.

In this review, we will discuss recent research progress in immunological and molecular characterization of CCA and GBC and its implication in the novel immuno- and targeted therapies, which hold promise in the future treatment of non-responding BTC patients.

## 2. Cholangiocarcinoma (CCA)

CCAs are a group of heterogeneous BTCs that can arise from cholangiocytes in the biliary epithelium and epithelial cells in peribiliary glands [13] or from progenitor cells or mature hepatocytes [14]. pCCA and dCCA are mucin-producing adenocarcinomas or papillary tumors that can develop either in the right or left hepatic duct (or both), at their junction [15], or in the common bile duct [16]. iCCA is a conventional CCA type that originates from intrahepatic bile ducts, and it is subclassified depending on its morphology and molecular characteristics [17]. Development of the iCCA is a multistep process of dysplastic and in situ lesions leading to cancer, which can be sometimes mistaken for intrahepatic metastases of gastric or pancreatic primary carcinoma [17]. iCCA and HCC have a similar morphology, risk factors, and oncogenic pathways; however, a correct distinction is necessary because the treatment strategies and prognosis are extremely different. CCA incidence rates vary among geographical regions, with higher rates in Eastern European and Asian countries [18]. Despite the differences in the incidence rates, the mortality rate is increasing worldwide [19]. Due to the complexity of CCA heterogeneity, classification, and later-stage detection, effective targeted molecular or immune therapies, alone or in the combinations with standard systemic drugs, are urgently needed.

### 2.1. Immunological Characterization of CCA Infiltrates 

Evidence for the use of checkpoint inhibitors as first- or second-line therapy in the medical management of patients with solid cancers has reshaped cancer treatment in 21st century [20,21]. The switch in the perspective from cancer to immune cell targeting turned immunotherapy into the fourth pillar of cancer treatment, after surgery, radiotherapy, and chemotherapy. In order to consider new immunological treatments for BTCs, and especially for CCA, it is important to characterize the tumor microenvironment (TME), which surrounds tumor epithelial regions and embeds the immune cells that could directly eradicate tumor.

In CCA, the TME is composed of blood vessels, signaling molecules, activated cancer-related fibroblasts (CAFs), and extracellular matrix (ECM), which form a rich desmoplastic tumor stroma [22]. In iCCA/pCCA the ECM is extremely rigid and favors tumor interactions with CAFs and immune cells [23]. It was previously demonstrated that the tumor stroma in CCA is composed of the abundant α-smooth muscle actin (α-SMA)^+^ myofibroblasts (CAFs) that outweigh the tumor itself [24] and block the infiltration of immune cells [25]. CAFs derive from hepatic stellate cells, portal fibroblasts, and probably bone-marrow-derived fibrocytes [26]. However, whether they originate from endothelial cells through endothelial-to-mesenchymal transition (EMT) is still unknown [27].

Besides the direct impact on tumor cells by regulating their proliferation, survival and migration, CAFs regulate angiogenesis and immunity by secreting a vast number of soluble chemokines and growth factors, such as chemokine (C-X-C motif) ligand 14 (CXCL14), interleukin 8 (IL-8), IL-13, vascular endothelial growth factor (VEGF), and fibroblast growth factor (FGF), that stimulate stromal cells and recruit inflammatory immune cells in tumor bed [26]. Recently, a study by Zhang et al. evaluated both iCCA and peritumoral samples to elucidate the comprehensive transcriptomic landscape and the intercellular communication network [28]. They identified six distinct fibroblast subsets, with a prevalent population of CD146^+^ vascular CAFs that highly express microvasculature signature genes and high levels of immunosuppressive IL-6. The latter one was strongly expressed by CAFs after the exposure to exosomal miR-9-5p secreted by iCCA. Moreover, another study characterized the composition and the function of the TME and performed a TME-based classification of 566 iCCA [29]. Based on the immune responses and signaling features (liver activity, inflammation, and immune resistance), they define four TME-based iCCA subtypes: 45% of iCCA displayed an immune desert “cold” phenotype, whereas the other subtypes differed in nature (lymphoid, myeloid, and mesenchymal) and abundance of tumor-infiltrating cells. Importantly, 11% of tumors showed the inflamed “hot” subtype with massive tumor-infiltrating lymphocytes (TILs) infiltration that could be targeted by immunotherapy.

#### 2.1.1. T and B Cells in CCA

In general, immune cells are classified into the two main groups of cells that show the innate and adaptive immune responses. Among them, the cytotoxic CD8^+^ T lymphocytes (CTL) and natural killer (NK) cells have the capacity to recognize the tumor and to elicit a tumor cell death via a release of cytotoxic granules [30,31]. To get in touch with tumor cells, T cells need to enter into the epithelial tumor zones. However, CTLs migrate poorly in dense matrix, and they preferentially migrate in regions of loose fibronectin and collagen surrounding the tumor [32] due to their capacity to crawl onto the collagen fibers [33]. It was shown that T cells need Myosin-IIA for the process of pushing their nucleus through the endothelium [34], and since they do not produce proteases that can degrade the ECM permitting cell migration through dense tissue, they are helped by the chemokines that guide them to the tumor regions. These data show that the density but also the orientation of collagen structures contribute to generally keeping T cells in the stroma, away from tumor cells.

With respect to CCA, CD8^+^ T lymphocytes have been studied in terms of presence, location, and some functionality within the tumor. New studies demonstrated the importance of CD8^+^ T cell specificity and cytotoxicity; however, an extensive phenotypic analysis of different subpopulations of CD8^+^ T cells is still missing. Based on an available immunohistochemical (IHC) staining, CD8^+^ T cells are the most abundant TILs inside the tumor regions [35]. Recently, it was shown that almost 70% of eCCA were infiltrated with CD8^+^ TILs, and the longer OS was observed in patients with tumors highly infiltrated with memory CD8^+^CD45RO^+^ TILs [36]. These results are in line with the observation that CD8^+^ TIL infiltrates correlate with better OS in BTC patients [37,38]. The highest infiltration with CD8^+^ TIL has been linked to a higher tumor mutational burden (TMB) in solid cancers [39,40], and despite the fact that CCA is not a highly mutated cancer, such as lung cancer or melanoma [41], several tumor-associated antigen (TAA)-derived epitopes were identified as candidate epitopes for immunotherapy [42].

Recently, a transcriptomic study of 10 iCCA demonstrated that the varying degree of heterogeneity in iCCA cells shapes the TME and decreases the cytotoxicity of CD8^+^ T cells. The authors indirectly link these results with the hypoxia-induced VEGF expression, as they speculate that the TME reprogramming by VEGF induction incites the restriction of T-cell infiltration by the transforming growth factor β (TGF-β) and provokes their lower responsiveness to immunotherapeutic treatments [43]. It is known that tumors often downregulate the expression of human leukocyte antigen (HLA) as a mechanism of the evasion from immune recognition and destruction by T cells [44]. However, contradictory results have been obtained in different cohorts of iCCA and eCCA, ranging from the description of high expression of HLA-I on tumor cells [45] to those with only 40–50% of HLA-I-positive tumors [46,47]. Nevertheless, regardless of the analyzed cohort, CD8^+^ T cells that infiltrate tumor zones have the greatest capacity to induce a tumor cell death. Further, expression of programmed death ligand 1 (PD-L1) has been analyzed, and its recurrent expression has been observed in multiple cohorts of iCCA and eCCA [46,47]. However, immune cells seem to express more PD-L1 than cancer cells [46,48,49,50,51], and PD-L1 expression on tumor cells correlates with a lower response to immunotherapy [52].

Interestingly, other TILs, the CD4^+^ T cells, stay in the peritumoral zones rather than tumor regions [35,53]. In a cohort of 306 CCA samples, the IHC staining demonstrated that high tumor infiltration with CD4^+^ TILs is a protective factor; indeed, it correlates with a longer patient OS [37]. Moreover, phenotypic characteristics of CD4^+^ T cells have been examined, especially of those harboring a Th1 response to the Erbb2 interacting protein (ERBB2IP) expressed by the CCA metastases [54]. In this study, it was demonstrated that the adoptive transfer of antigen-specific Th1 cells decreased the size of target lesions and prolonged the stabilization of disease in one patient. 

On the contrary, very little is known about the regulatory T cells (Treg) in CCA. It is well admitted that the interplay between TILs and innate immune cells impacts the antitumor immune response. In that light, an elevated neutrophil-lymphocyte ratio (NLR) in iCCA was correlated with poor OS and low recurrence-free survival and associated with a higher percentage of PD-1^+^ CD4^+^ and CD8^+^ TILs, as well as IFN-γ^+^ TILs [55]. Finally, B cells, have also been poorly examined in CCA, and very little evidence is available; it has been reported that B cells correlate with a favorable outcome. However larger studies are needed to confirm their role [35,37].

Overall, these studies show that the characterization of TIL subpopulations, their functionality, and the implication in antitumor immune response in CCA are still underexplored, and we speculate that the increase in CCA incidence will encourage novel research programs in the near future.

#### 2.1.2. Innate Immune Cells

Innate immune cells, such as tumor-associated macrophages (TAM), tumor-associated neutrophils (TAN), myeloid-derived suppressor cells (MDSC), and dendritic cells (DC), shape the microenvironment of CCA by manifesting mostly immunosuppressive and protumorigenic phenotypes.

TAM are abundant cells with M2 phenotype, with high phagocytic activity involved in the matrix remodeling and angiogenesis [56,57]. In CCA, the CD14^+^/CD16^+^ TAM population derives from circulating monocytes and not from Kupffer liver cells [58,59] and they are attracted to the CCA TME by the chemokine (C–C motif) ligand 2 (CCL2) released by CAFs [60], TAN and liver-residing macrophages [61]. CCA cells also participate in M2 TAM polarization by secreting IL-6 and TGF-β, together with IL-10 and colony-stimulating factor 1 (CSF1) [62]. Moreover, it was shown that CCA stem-like cells induce M2 polarization by the secretion of IL-13 and IL-34 [63,64]. In iCCA and eCCA, higher TAM infiltration has been associated with a poor survival [38,62]; however, the presence of CD68^+^ TAM in the invasive front of iCCA correlated with a reduced disease recurrence [65].

Similarly, the presence of CD66^+^ or CD15^+^ TAN in CCA has been correlated with the worst patient outcome [66,67,68]. In vitro experiments showed that the chemoattraction of TAN is mediated by the phosphoinositide 3-kinase-protein kinase B (PI3K-Akt) and the extracellular signal-regulated kinase 1/2 signaling pathways [69], which was confirmed in animal models.

On the contrary, MDSCs are a heterogeneous group of cells derived from immature myeloid progenitors, with very strong immunosuppressive properties [70]. They are recruited to the TME mainly by CAFs, through the fibroblast activation protein (FAP) in CCL2/STAT3-dependent manner, but also via the secretion of IL-6 and indoleamine 2,3-dioxygenase (IDO) [71,72]. They synthesize reactive oxygen species (ROS) and produce enzymes like arginase, inducible NO synthase, cyclooxygenase 2 (COX2) and IDO, as well as the cytokines TGF-β and IL-10, which are all involved in the immunosuppression of effector cytotoxic T cells [73].

Moreover, TGF-β and IL-10 can impair the cross-presentation of DCs to T cells in multiple cancers and it has been shown that the suppression of TGF-β and IL-10 receptors on self-differentiated DCs enhanced the activation of effector T cells against CCA tumor cells [74]. In the same light, another study showed that the self-differentiated monocyte-derived DC presenting cAMP-dependent protein kinase type I-alpha regulatory subunit (PRKAR1A) had the highest ability to induce robust cytotoxic T cell response against CCA cells [75]. Furthermore, the decreased number of circulating myeloid DCs and monocytes positive for the Fc fragment of IgE high-affinity receptor I (FcεRI), as well as the decreased frequency of tumor necrosis factor alpha (TNFα)-proinflammatory DCs that have been observed in CCA patients, could functionally affect DC-mediated immune responses [76]. Regarding DC positioning inside the tumor zones, as is the case with T cells, mature CD83^+^ DCs stay predominantly outside of the tumor center, with immature CD1a^+^ DCs recruited into the tumor [53].

On the contrary, NK cells are cytotoxic innate cells with the capacity to destroy tumor cells in an antigen-nonspecific manner [77], but less is known about the role of NK cells in CCA. However, in vitro and in vivo studies demonstrated that the endogenous iCCA CXCL9 correlates with the higher infiltrations of NK cells in the tumor zones [78] and that the biliary epithelium could present antigens to and activate NK T cells [79].

All these studies demonstrate that, as it was a case with adaptive immune cells, the innate cells are still underexplored in CCA, and a more profound analysis is yet to come.

#### 2.1.3. Immunotherapeutic Strategies in CCA

Strategies to enhance the efficacy of immunotherapy need to take into account the immune escape mechanisms exploited by cancer. In CCA, tumor cells are a reservoir of protumorigenic and immunosuppressive molecules, such as IDO, IL-10, and TGF-β [61,80], which participate in the immunosuppression of the effector CTL and the attraction of innate immune cells as TAN, TAM, MDSC, and Treg. Moreover, they secrete IL-6, which polarizes the macrophages into M2 phenotype and attracts MDSCs [81]. Clinical trials targeting IDO are ongoing in different solid cancers, such as melanoma, lung, and renal cancer [82], and despite the controversy regarding the side effects [83], promising results have been obtained [84]. We speculate that the future clinical trials in CCA could include IDO inhibitors alone or in the combination with anti-PD-1, as well as other anti-TGF-β antibodies, which were tested in preclinical settings and demonstrated an unleashed anti-tumor CTL response upon TGF-β blockade, rendering tumors more susceptible to anti-PD-(L)1 [85] and leading to tumor regression. 

A dense matrix and the secretion of CXCL12 by CAFs [86] actively play a role in the exclusion of T cells, limiting their contact with tumor cells and the cytotoxic potential. A rich desmoplastic stroma composed of CAFs in CCA is a potential target for optimizing the therapeutic strategies. Multiple trials targeting their activation and/or action are ongoing in several cancers [87]. 

Tumor cells can also directly bypass T cell recognition by the expression of PD-L1 and/or downregulating the HLA-I expression. In CCA patients, PD-L1 seems to be more expressed on CD8^+^ T cells than on tumor cells and correlates with the lower infiltration of CTL in tumors and a worse patient survival [46,88]. In a preclinical model of an acute viral infection, the higher expression of PD-L1 on CD8^+^ T cells decreased the proliferation, functional maturation, and production of multiple granzymes of the effector CTL [89], similar to the model of graft-versus-host disease (GVHD), where Pdl1^–/–^ CD8^+^ T cells diminished the production of inflammatory cytokines and enhanced the rate of CTL apoptosis [90]. These data implicate that the use of anti-PD-L1 antibodies in CCA will primarily modulate the functionality of CTLs rather than block the interaction between PD-1/PD-L1 on CTL and tumor cells. Importantly, HLA-I seems to be downregulated in some CCA patients, but the normal expression is recorded and correlated with a better patient outcome [46]. 

**Figure 2:**
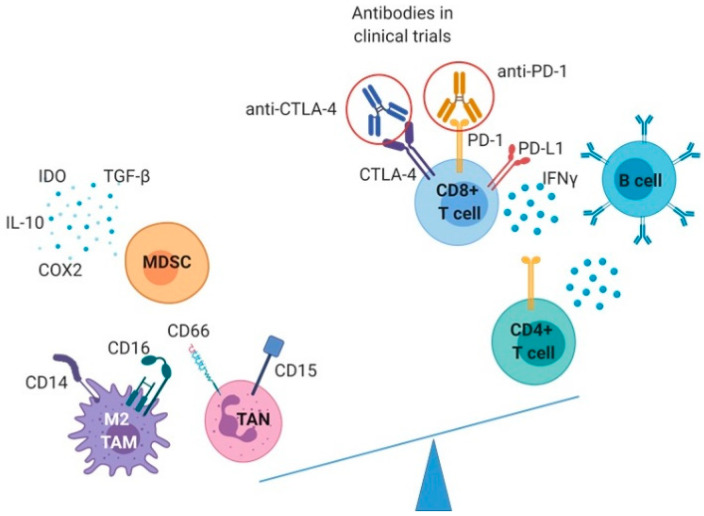
Immunological characterization and immunotherapeutic strategies in CCA. Schematic representation of the innate immune cells promoting tumor growth (left part of the balance) in comparison with the adaptive immune cells implicated in the tumor growth control. Abbreviations: COX2: Cyclooxygenase 2; CTLA-4: Cytotoxic T-lymphocyte antigen 4; IDO: indoleamine 2,3-dioxygenase; IFNγ: Interferon γ; IL-10: Interleukin 10; MDSC: Myeloid-derived suppressor cell; TAM: Tumor-associated macrophage; TAN: Tumor-associated neutrophil; TGF-β Transforming growth factor beta; PD-1: Programmed death 1; PD-L1: Programmed death ligand 1.

Despite a higher infiltration with the immunosuppressive innate cells, the reported infiltration with CTL encouraged the conduction of clinical trials targeting inhibitory receptors, such as programmed death 1 (PD-1) and cytotoxic T-lymphocyte antigen 4 (CTLA-4), which are already successfully administered in multiple solid cancers (Figure 2). 

At this moment, 35 clinical trials in Phases 1–3 targeting PD-1 and/or CTLA-4 alone or in the combinations with other chemotherapeutic drugs have been registered as ongoing on ClinicalTrials.gov, mainly in China and the US, with two trials in Europe (Germany). Among all the studies, one study demonstrated the complete disappearance of liver lesions in one patient after the treatment with nivolumab (anti-PD-1) plus lenvatinib (multiple kinase inhibitor against VEGFR1, VEGFR2, and VEGFR3 kinases), and with the lung metastases stable after 9 months of therapy [91]. Other results from these ongoing clinical trials are highly expected by the medical community, due to the inadequate therapeutic options in CCA treatment and a very low patient survival.

We speculate that the era or immunotherapy will bring promising results in the treatment of CCA, as was the case for multiple other solid cancers.

### 2.2. Molecular Characterization of CCA

With the development of the powerful genetic screening techniques, multiple genetic driver mutations have been identified in different subtypes of CCA in order to respond to the unmet medical need for the request of novel prognostic and/or predictive biomarkers of targeted therapies. Before the consensus in CCA subtype nomenclature was established, a small cohort of 40 Caucasian patients with eCCA was analyzed, and a longer OS was observed in patients with a single-nucleotide polymorphism (SNP) at a position 825 C > T in *GNB3* gene [92]. More recently, another study found that *KRAS*, *TP53, ARID1A,* and *SMAD4* are the most prevalently mutated genes in eCCA. Based on the transcriptomic data, the authors classified 189 eCCA into four subgroups depending on the genes that were expressed: the metabolic, proliferating, mesenchymal, and immune group. Moreover, they identified 25% of tumors with actionable genomic alterations, which could be potentially targeted in a therapeutic setting [50].

On the contrary, a study in 119 iCCA patients showed that iCCA are phenotypically different from eCCA, and those iCCA tumors can be classified into the inflammation and proliferation group, with distinct characteristics and genes activated [93]. In general, several other gene variants have been correlated with a higher risk of CCA development [94], such as *EZH2* [95], *NRF2* [96], *XRCC1* [97], *ABCB2* [98], *ATP8B1* [99], and *NKG2D* [100]. Capital work was done by Nakamura et al., who analyzed 231 CCA and 29 GBC of Japanese patients by whole-exome (WES) and transcriptome (RNASeq) sequencing to compare differences among BTCs, which uncovered the genomic alterations that included new potential therapeutic targets [88]. The authors found a significantly lower number of somatic mutations in CCA than in GBC and described new driver fibroblast growth factor receptor 2 (*FGFR2*) fusion genes, exclusively detected in iCCA, to complete the list of already-described gene fusions [101,102]. Moreover, other fusion genes have been discovered for the first time, such as *ATP1B-PRKACA* and *ATP1B-PRKACB*, exclusively present in pCCA/dCCA [88]. Otherwise, clusters of missense mutations were observed in 6 oncogenes *KRAS*, *PIK3CA*, *IDH1*, *NRAS*, *GNAS,* and *ERBB2*, with *KRAS* mutations more prevalent in pCCA/dCCA than in iCCA [103].

#### Molecular Therapeutic Targets

Genetic screening has led to the discovery of almost 40% of uncovered potentially targetable genetic mutations, which implies that a diverse genetic composition depends on the anatomical localization of the biliary cancers and thus emphasizes the need for the personalized medicine and genomic analyses of the tumor samples in clinical disease management.

A recent multicenter, randomized, double-blind, placebo-controlled, Phase 3 study evaluated the efficacy and safety of ivosidenib, a small inhibitor of mutated *IDH1* gene in patients with previously treated *IDH1*-mutant iCCA. This study showed that patients had a clinical benefit and an increased progression-free survival (PFS) in the experiment group, which was 2.7 months compared to 1.4 months in the control group [104].

As registered on ClinicalTrials.gov, no fewer than 40 trials in Phases 1–4 in China, Japan, the US, and Europe are testing the inhibitors of fibroblast growth factor receptor (FGFR), epidermal growth factor receptor (EFGR), Janus kinase (JAK), VEGFR2, and/or poly (ADP-ribose) polymerase (PARP) alone or in the combination with other systemic chemotherapeutic drugs. Moreover, there is a rationale for *KRAS*-mutant CCA treatment by MEK inhibition. A Phase 2 study of selumetinib use in metastatic BTC patients demonstrated a median PFS of 3.7 months and a median OS of 9.8 months [105]. Additionally, the treatment with the oral BRAF inhibitor vemurafenib led to a partial response in one patient among eight patients with *BRAF V600*-mutated CCA, even though *BRAF* mutations predominantly occur in iCCA at low frequency (3–5%) [106,107,108].

Recently, the FDA approved the use of pemigatinib for the treatment of adults with previously treated, unresectable locally advanced or metastatic CCA with the FGFR2 fusion or other rearrangements, and it is the first targeted therapy for CCA patients [109]. FGFR2 fusion is exclusively present in iCCA patients, and pemigatinib got the approval based on the results of the FIGHT-202 study (NCT02924376). It was a multi-center, open-label, single-arm study, which included 146 patients from 2017 to 2019. The overall response rate (ORR) was 36% with a median of 9.1 months of the duration of response [110]. Since pemigatinib was classified by the FDA as an Orphan Drug, it was approved under an accelerated process. Besides this approval, other therapeutic options could be considered, since it was demonstrated that the inhibition of heat-shock protein 90 (HSP90) is an alternative to direct FGFR-kinase inhibition in FGFR2-fusion-driven cancers. HSP90 is a molecular chaperone implicated in the cellular housekeeping functions, such as protein folding, mediating post-translational protein homeostasis, and maintenance of an oncoprotein stability [111]. The selective HSP90 inhibitor ganetespib induced the loss of fusion protein expression, the inhibition of oncogenic signaling, and a consequent cancer-cell cytotoxicity in FGFR-fusion-driven bladder cancer [9].

**Figure 3:**
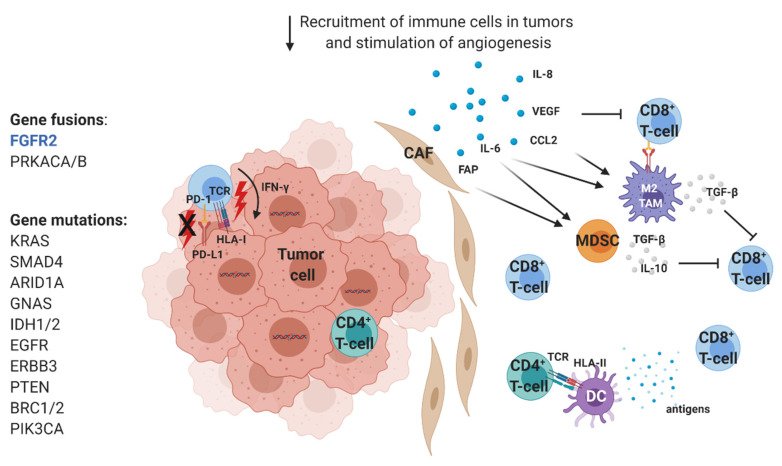
Molecular and immunologic characterization of BTCs. CAF: Cancer-associated fibroblast; CCL2 Chemokine (C–C motif) ligand 2; DC: Dendritic cell; FA: Fibroblast activation protein; HLA-I: Human leukocyte antigen I; HLA-II: Human leukocyte antigen II; IL-6: Interleukin 6; MDSC: Myeloid-derived suppressor cell; PD-1: Programmed death 1; PD-L1: Programmed death ligand 1; TAM: Tumor associated macrophage; TCR: T-cell receptor; TGF-β Transforming growth factor beta; VEGF Vascular endothelial growth factor.

To summarize, with one targeted therapy drug approved and multiple clinical trials ongoing, there is a rationale to expect novel therapeutic options for CCA patients in the close future (Figure 3).

## 3. Gallbladder Cancer 

GBC is a rare aggressive adenocarcinoma of the biliary tract, and it constitutes 1.2% of all cancer deaths. It develops from the mucosal lining of the gallbladder [112] most prevalently in women, patients older than 65 years, and Native Americans, mainly upon the chronic inflammation caused by the presence of gallbladder stones. Gallbladder stones represent the central risk for GBC development; however, only 1 in 200 people with gallstones will develop GBC [113,114]. In general, the link between the inflammation and GBC has been previously established in other inflammation-related conditions, such as diabetes, obesity, and the infections with *Helicobacter* species [115,116,117].

Despite the advances in hepatobiliary imaging techniques, the disease is discovered at an advanced stage, and half of the newly diagnosed GBC are diagnosed after cholecystectomy for a presumed benign disease [118]. The 5-year survival of patients with locally advanced disease without the metastases spreading is around 85%, contrary to those patients with stages III–IV, whose life expectancy is only 3–8%. General symptoms of GBC include the loss of appetite, abdominal pain, and weight loss. These symptoms include itching (pruritus) and jaundice [113].

Treatment of GBC patients relies on a surgical resection. It is the only potentially curative option for GBC, but the main problems are frequent locoregional and/or distant recurrences even after a complete resection [119,120]. Due to its rarity, GBC patients were historically included in combined BTC trials, so other treatment regimens, besides a surgical resection, are similar to those for CCA. First-line treatment for GBC patients is a combination of the chemotherapy drugs gemcitabine and cisplatin, based on the results of the ABC-02 trial [121]. More recently, a combination of folinic acid, fluorouracil, and oxaliplatin (FOLFOX) is used as a second-line treatment option, based on the results of the ABC-06 trial, which demonstrated that a median OS was higher in the FOLFOX arm (6.2 months) compared to the supportive-care-only arm (5.3 months) [122].

To expand the panel of potential therapeutic options, novel chemotherapeutic combinations have been tested in BTC patients, including the GBC. An open-label, single-arm Phase 2 trial evaluated the efficacy of fluorouracil, leucovorin, irinotecan plus oxaliplatin (FOLFIRINOX), with two different dosages in 40 patients who had disease progression or unacceptable toxicity after more than three cycles of gemcitabine plus cisplatin [123]. The results showed that FOLFIRINOX is a safe combination and could be considered as a treatment option. Nevertheless, a new era of precision medicine drugs and immunotherapy is evaluated in clinical trials for GBC patients, however, to a lesser extent than for other BTCs, probably due to its rare onset. With the increasing rate of GBC and due to the novel genomic studies that shed light on the innate differences in GBC development and spreading in comparison with other BTCs, novel therapeutic options are highly expected.

### 3.1. Molecular Characteristics and Immune Infiltrates in GBC

In the past decade, the genome profiles of a large number of patients included in the international BTC cohorts have been analyzed via next-generation sequencing, and they identified the unique molecular subtypes, mutations, and genomic rearrangements. The largest cohort analyzed 57 cases and found that the most mutated genes were *TP53* (47%), *KRAS* (78%), and *ERBB3* (12%) [124]. Moreover, *TP53* was the most frequently mutated gene across multiple GBC studies [88,124,125], whereas *KRAS* is among the most frequently mutated genes in several solid cancers.

**Figure 4:**
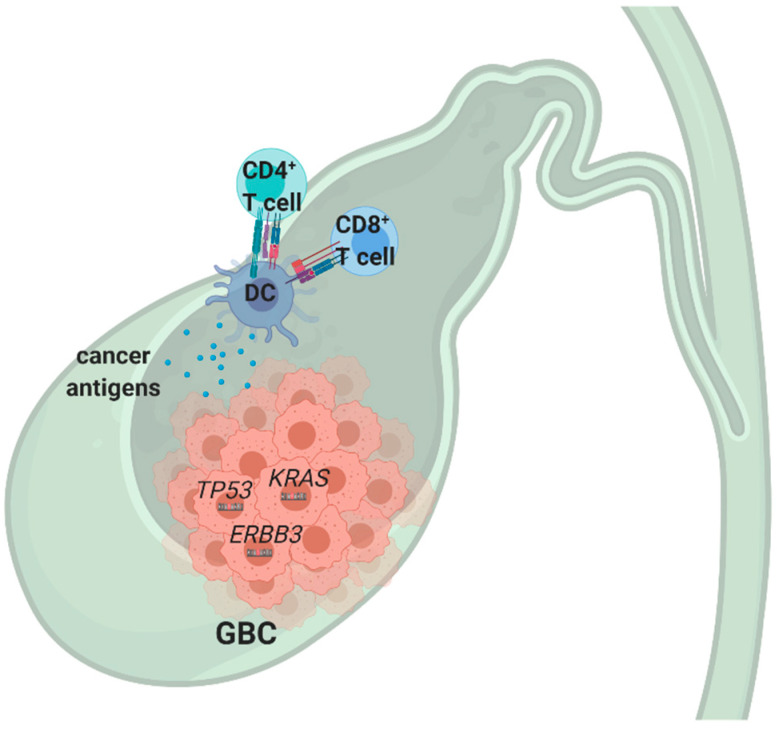
Molecular characterization of recurrent mutations and immune infiltrates in GBC. Abbreviation: DC: Dendritic cell.

Contrary to these results, a recent review by Valle et al. combined the results from multiple studies and found a lower frequency of *KRAS* mutations in GBC, which were prevalent in only 8–13% cases [126]. In general, *KRAS* mutations can only be targeted indirectly by blocking BRAF, MEK, and ERK. In a Phase 2 trial combining the use of BRAF and MEK inhibitors, the patients had an increased clinical benefit, with both median PFS and OS increase [127]. Regarding the *ERBB3* gene, a dominant prevalence of C > T mutations were found, with ErbB signaling the most extensively mutated pathway, affecting more than 1/3 of analyzed patients, which was correlated with the worse outcome [124]. In addition to these findings, *PIK3CA* mutations were found in one cohort [128], whereas no mutations have been identified in *IDH1/2* genes in GBC, contrary to CCA [129]. Regarding the microsatellite instability (MSI), which was previously linked to a better responsiveness to immunotherapy in several cancer types [130,131], only 6% was present in the GBC [132,133] (Figure 4).

Taken together, no molecular classification of GBC has been published thus far. GBC cohorts are rare due to a limited number of GBC patients; however, with the rise of the molecular studies aiming to better depict GBC, we speculate that the molecular classification will follow to better select the patients who could benefit from potential second-line options.

Importantly, very little is known about the immune infiltrates in GBC. A recent study evaluated the feasibility of the production of the effective DCs using heat-conditioned cell lysates derived from GBC cell lines. These in vitro studies demonstrated that conditioned lysate-matured DCs strongly induced the activation of CD4^+^ and CD8^+^ T cells in both allogeneic and autologous cell co-cultures. Moreover, in vitro stimulated CD8^+^ T cells were able to recognize HLA-matched GBC cell lines, demonstrating efficient priming of DCs by cancer cells and a subsequent presentation of antigens to T cells [134]. These results opened the way for future immunotherapy approaches; however, other studies are needed to characterize immune infiltrates. In addition, the studies demonstrating the interactions of TILs with GBC cells are missing, which could encourage new clinical trials.

### 3.2. Recent Advances in Targeted Therapy and Immunotherapy of GBC

Clinical trials evaluating the efficacy of new combinations of molecular drugs targeting mutated pathways with the approved chemotherapies and/or immunotherapy plus chemotherapy are ongoing or recently terminated in the US and China. The use of afatinib or regorafenib (tyrosine-kinase inhibitors, TKI), cetuximab or erlotinib (anti-epidermal growth factor receptor; EGFR), sorafenib or bevacizumab (anti-VEGF), trastuzumab (anti-ErbB2) or cobimetinib (MEK inhibitor) in a combination with the chemotherapy are or were investigated in GBC patients. Trials evaluating anti-VEGF and anti-EGFR drugs ended, showing that treatment with sorafenib did not result in an objective response [135]. Further, the combination of sorafenib and erlotinib did not improve the survival of an unselected group of BTC patients [136].

A meta-analysis of seven randomized controlled trials showed that anti-EGFR drugs increased PFS but without the statistically significant difference in the overall PFS between the combination regime and a chemotherapy alone [137]. Results from the trials including the immunotherapeutic drugs, avelumab, durvalumab, or atezolizumab (anti-PD-L1) are being evaluated. Moreover, a large, Japanese, non-randomized, multicentre, open-label Phase 1 study in BTCs (including GBC) evaluated the use of nivolumab (anti-PD-1) alone or in the combinations with cisplatin plus gemcitabine in unresectable or recurrent BTCs. The results showed a good safety profile with signs of a clinical activity, but the authors conclude that larger randomized studies (Table 1) are needed to provide more evidence for the clinical use [138].

## 4. Conclusions

BTCs are a rare group of cancers that arise throughout the biliary tree. For that reason, they were historically classified in the same treatment or experimental group, but increasing evidence ratifies the molecular and immunological differences between CCA and GBC, as well as the differences in their TME. In the last decade, an extensive molecular characterization demonstrated that the mutations in *KRAS*, *SMAD4*, *ARID1A*, *GNAS*, and *IDH1/2* gens as well as FGFR2 fusions have been associated mostly with CCA, whereas *EGFR*, *ERBB3*, and *PTEN* mutations are prevalent in GBC. As some of these mutations/fusions are possible medical targets, this knowledge shaped clinical trials, which are still represented in a modest number compared to other cancer types, such as lung or breast cancer.

Recently, an anti-FGFR2 antibody has been approved as a second-line treatment option in iCCA, and it is the first targeted therapy for this cancer [109]. Since the molecular heterogeneity, etiology, and epidemiology are the hallmarks of CCA, we presume that the genetic differences will probably be routinely tested in a close future with the rise of a personalized medicine, in order to better select patients that could benefit from novel drugs. Moreover, with the rise of the extremely promising results of immunotherapy in solid cancers, both in preclinical and clinical trials, including autologous cell transfer, vaccination, and immune checkpoint inhibitors, new perspectives have been given in addition to the therapies targeting molecular mutations. CCA is no exception to the immune escape, and due to its rigid stroma and highly active CAFs, the immune cells stay predominantly at the tumor margin. These observations are mainly based on the IHC staining, but new studies are increasing in number and are starting to delineate immune cell populations and their functions responsible for the cancer escape.

To conclude, we expect new exciting results coming from the ongoing clinical trials, including immunotherapeutic drugs checkpoint inhibitors anti-PD-(L)1 and anti-CTLA-4, and we believe that other immunosuppressive molecules will be targeted in future trials, based on the promising results from different solid tumors.

## Figures and Tables

**Table 1:** Selected Ongoing Clinical Trials in BTCs Targeting Molecular Mutations/Fusions And Using Immune Checkpoint Inhibitors Alone or in the Combinations.

Type of Treatment	Target	Pathway	Trial Type (Number of Patients)	Agent	Clinicaltrails.Gov Reference
Molecular targets		Respiratory electron transport	Phase 2 (36)	Olaparib	NCT04042831
	ARID1A	ATP synthesis by chemiosmosis coupling			
		Heat production by uncoupling proteins			
		Oxytocin signaling pathway	Phase 2 (68)	Varlitinib	NCT03231176
		Direct p53 effector	Phase 2/3 (490)	Varlitinib	NCT03093870
	EGFR		Phase 1/1b (48)	Afatinib	NCT02451553
			Phase 2 (6452)	Afatinib	NCT02465060
			Phase 1/2 (25)	Erlotinib	NCT02273362
	PTEN	Metabolism of proteins	Phase 2 (6452)	Taselisib	NCT02465060
		Direct p53 effector			
	BRCA1	Metabolism of proteins	Phase 1/2 (110)	Rucaparib	NCT03337087
		ERK signaling			
		Angiogenesis	Phase 2 (143)	Derazantinib	NCT03230318
		Wound healing			
	FGFR2	Cell migration			
		Neural outgrowth			
		Embryonic development			
		RET signaling	Phase 2 (57)	RC48-ADC	NCT04329429
	ERBB2/HER2		Phase 2 (15)	Trastuzumab	NCT03613168
			Phase 2 (100)	Trastuzumab	NCT03185988
			Phase 1/2 (82)	A166	NCT03602079
	PARP	Post-translational modification	Phase 2 (35)	Rucaparib	NCT03639935
			Phase 1/2 (110)	Rucaparib	NCT03337087
	MEK	Oxytocin signaling pathway	Phase 2 (57)	Selumetinib	NCT02151084
			Phase 3 (50)	Sorafenib	NCT04163237
			Phase 1 (17)	Sorafenib	NCT02292173
Checkpoint inhibitors	PD-1	T-Cell receptor and co-stimulatory signaling	Observational study (100)	Nivolumab/ pembrolizumab	NCT03695952
		Class I MHC mediated antigen processing and presentation	Phase 2 (30)	Nivolumab	NCT04057365
			Phase 1/2 (40)	Nivolumab	NCT03785873
			Phase 3 (200)	Toripalimab	NCT03949231
	PD-L1	Class I MHC mediated antigen processing and presentation	Phase 2 (90)	Durvalumab	NCT02821754
		Cell adhesion molecules	Phase 2 (50)	Durvalumab	NCT04238637
	CTLA-4	T-Cell receptor and co-stimulatory signaling	Phase 2 (45)	Ipilimumab	NCT03222076
		Class I MHC mediated antigen processing and presentation			
